# Malaria Vectors’ Diversity and Seasonality in Sustaining Disease Transmission in an Endemic Area of Faladie, Mali

**DOI:** 10.3390/tropicalmed11080216

**Published:** 2026-08-03

**Authors:** Fatalmoudou Tandina, Safiatou Niare Doumbo, Moussa Djimdé, Sékou Sissoko, Privat Agniwo, Amagoron Mathias Dolo, Abdrahamane S. Kamaté, Amatigue Zeguime, Salif Yirampo, Boucary Ouologuem, Aichata Dembélé, Hawa Dembélé, Mohamed Touré, Abdoulaye Kaloga, Francois Dao, Siaka Goita, Mamadou M. Tekete, Mahamadou A. Thera, Abdoulaye K. Koné, Abdoulaye A. Djimdé, Laurent Dembele

**Affiliations:** 1 Parasites & Microbes Research and Training Center (PMRTC), Faculty of Pharmacy, Université des Sciences, des Techniques et des Technologies de Bamako (USTTB), Point G, Bamako P.O. Box 1805, Mali; sdoumbo@icermali.org (S.N.D.); mdjimde@icermali.org (M.D.); sissoko.sekou@icermali.org (S.S.); privatagniwo@yahoo.com (P.A.); mathiasdolo@icermali.org (A.M.D.); abdrahamanekamate14@gmail.com (A.S.K.); amatigue@icermali.org (A.Z.); salif.yirampo@icermali.org (S.Y.); ouologuem@icermali.org (B.O.); aichatabougougnire@gmail.com (A.D.); hawa.dembele@icermali.org (H.D.); mohamedcheicktoure1@gmail.com (M.T.); kalogadolan111@gmail.com (A.K.); francoisd@icermali.org (F.D.); sgoita@icermali.org (S.G.); mtekete@icermali.org (M.M.T.); mthera@icermali.org (M.A.T.); fankone@icermali.org (A.K.K.); adjimde@icermali.org (A.A.D.); 2 Centre de Recherche pour la Lutte Contre les Maladies Infectieuses Tropicales (CReMIT/TIDRC), Université d’Abomey-Calavi, Abomey-Calavi 03, Cotonou 4177, Benin

**Keywords:** *Anopheles*, malaria, transmission, Faladie, seasonality, diversity

## Abstract

The widespread circulation of the *Anopheles* vector is observed during the rainy season and clearly decreases during the dry season, when only a few mosquitoes are able to survive. This study aimed to identify the major species sustaining continued malaria transmission across seasons. This cross-sectional study was conducted in a malaria-endemic area. Mosquitoes were captured in Faladie using pyrethroid spray catches during rainy and dry seasons. The collected mosquitoes were identified using morphological keys, combined with molecular techniques such as PCR-RFLP (IGS regions) for *Anopheles (An.) gambiae s.l.* identification. *Plasmodium* spp. positivity in each mosquito was characterized by qPCR using parasite 18 S genes. A total of 1082 and 1705 mosquitoes were captured inside the same houses in the rainy and dry seasons, respectively. In the rainy season, 454 (41.9%) were *Anopheles*, 624 (57.7%) *Culex* and 4 (0.4%) *Aedes*. During the dry season, 65 (3.81%) mosquitoes were *Anopheles*, while 1,640 (96.19%) were *Culex.* Morphologically, the anopheline population consisted of 100% *An. gambiae s.l*. The *Anopheles* species diversity in the rainy season comprised the sister taxa, *An. gambiae s.s.* and *An. Coluzzii,* followed by an *An. gambiae/An. coluzzii* hybrid and *An. arabiensis*. However, *An. coluzzii* was found as the only vector across the rainy and dry seasons that sustained malaria transmission. *Plasmodium falciparum* infection rates were 9.7% and 1.7%, respectively, for the rainy and dry seasons. *An. coluzzii* species can survive both the dry and the rainy seasons and sustain malaria through cross-seasonal transmission. This highlights the importance of tackling *An. coluzzii* species and residual malaria transmission to eradicate the disease in endemic areas.

## 1. Introduction

Malaria remains a major public health problem despite numerous control measures. The World Health Organization (WHO) estimated that there were 280 million cases of malaria and 600,000 deaths from the disease worldwide in 2024 [1]. Mali is one of eleven countries where malaria accounts for 64% of global malaria cases and 68% of deaths [1]. Mali is one of three African countries that introduced the malaria vaccine in 2025 to improve prevention of this disease [1].

In Mali, four strata of malaria transmission have been described: very-low-transmission areas (fewer than 100 cases per 1000 person-years), low-transmission areas (between 100 and 250 cases per 1000 person-years), moderate-transmission areas (between 250 and 450 cases per 1000 person-years), and high-transmission areas (450 or more cases per 1000 person years) [2]. These four strata of malaria transmission are mainly linked to the seasonal transmission of malaria, where heavy transmission occurs in the rainy season, while the dry season has low disease transmission.

Faladie is a rural area with a high transmission rate, where malaria remains hyperendemic. Three *Plasmodium* species are found: *Plasmodium falciparum (P. falciparum)*, the most dominant species, followed by *P. malariae* and *P. ovale*. All three are responsible for malaria-related morbidity and mortality [3,4]. In this village, malaria is seasonal and follows the rainy season (from July to December), with a transmission peak in October. Locally, the highest asexual stage carriage occurs in October while the highest gametocyte carriage occurs in November. Natural decreases in the parasite’s gametocytes carriage and transmission have been observed during the dry season mainly from February to June [3].

*Anopheles* mosquitoes are vectors of the *Plasmodium* parasite; their distribution and malaria transmission competency depend on the ecological conditions of each region [5]. In Mali, twenty-eight species of *Anopheles* have been identified in various entomological studies [6]. *Anopheles coluzzii* Coetzee & Wilkerson, 2013, *An. gambiae s.s.* Giles, 1902, *An. arabiensis* Patton 1905, and *An. funestus* Giles, 1900, mosquitoes are the dominant vector species transmitting *Plasmodium* parasites in Mali [6,7]. Wide circulation of the vector is observed during the rainy season and clearly decreases during the dry season, with only a few mosquitoes able to survive during this non-optimal period [8]. However, the proportion of *Anopheles* mosquitoes that remain capable of transmitting malaria must be determined. The aim of this study is to evaluate the profile of the mosquito population associated with malaria transmission during the rainy versus dry seasons.

## 2. Materials and Methods

### 2.1. Study Sites

This study was conducted in Faladie, a rural capital of the Ntjiba commune located in the constituency of Kati in the region of Koulikoro in Mali. This rural area is situated 80 km from the Malian capital Bamako. According to the local registers, the commune of Ntjiba has 23 villages with an estimated population of 23,000 inhabitants. In this area, malaria is hyperendemic, and transmission is seasonal. High transmission occurs from July to January, while February to June corresponds to the low-transmission season [3,4].

### 2.2. Mosquito Collection and Morphological Identification

Adult mosquitoes were collected inside the concessions using pyrethroid spray catches (PSC) from September to October 2024 during the rainy season and from April to May 2025 during the dry season. All information relating to the collection of mosquitoes, e.g., the date and time of collection, village, house number/name of head of household, and geo-location, was noted on the mosquito collection sheets.

Mosquitoes were morphologically identified using the illustrated dichotomous identification keys for Robert et al. [9]; sex and gonotrophic state were also determined. After identification, only *Anopheles* mosquitoes were dissected to separate different parts (legs, abdomen and heads/thorax), and then stored at −20 °C.

### 2.3. Anopheles Gambiae Complex Species Identification by PCR-RFLP

PCR-RFLP was used to identify *An. gambiae* complex species. DNA extractions from individual mosquito heads/thorax samples were performed using the DNeasy Blood & Tissue Kit (Qiagen, Hilden, Germany) according to the manufacturer’s recommendations. PCR-RFLP (IGS regions) was used for *An. gambiae s.l* mosquito identification, as described by Fanello et al. [10], with the following modifications. A PCR mix final volume of 25 μL contained 5 μL of template DNA, 2.5 μL Buffer10× (New England Biolabs, Ipswich, UK), 2.5 μL MgCl_2_ (New England Biolabs, Ipswich, UK), 2.5 μL dNTP (New England Biolabs, Ipswich, UK), 2.2 μL primer UN (5′-GTGTGCCCCTTCCTCGATGT-3′), 2.8 μL GA (5′-CTGGTTTGGTCGGCACGTTT-3′), 1.6 μL AR (5′-AAGTGTCCTTCTCCATCCTA-3′), 5.775 µL H_2_O (Nuclease free water) and 0.125 μL DNA polymerase Taq (New England Biolabs, Ipswich, UK,). The PCR reaction was carried out with an initial step of incubation for 10 min at 94 °C to activate the DNA polymerase followed by 30 cycles. Each cycle consisted of denaturation for 30 s at 94 °C, annealing for 30 s at 50 °C and denaturation for 30 s at 94 °C, annealing for 30 s at 50 °C and extension for 30 s at 72 °C. The final cycle products were extended for 7 min at 72 °C. After amplification, 0.625 μL of Hha I (New England Biolabs, Ipswich, UK) in 10× rCutSmart^TM^ Buffer (New England Biolabs, Ipswich, UK) were added to the PCR reactions, and digestion was done at 37 °C for 3 h. Digested fragments were run through a Safe View^TM^ (Applied Biological Materials Inc., Richmond, BC, Canada) 2% agarose gel and photographed under light illumination.

### 2.4. Real-Time PCR for Malaria Infectivity Detection in Anopheles

Real-time PCR (qPCR) targeting the small subunit RNA gene (18S rRNA) was used in the speciation of the five human malaria parasites (*P. falciparum*, *P. malariae*, *P. vivax*, *P. ovale curtisi* and *P. ovale wallikeri*). qPCR was performed using primers, probes, and reaction conditions described previously by Lee et al. [11] and Potlapalli et al. [12], with minor modifications. The primers and probes used are listed in Table 1. The concentration for each reaction was 100 nM. Briefly, the multiplex assays for *Plasmodium* speciation were performed in a final volume of 10 μL containing 3 μL of template DNA, 5 μL of Multiplex PCR master mix (IDT, Leuven, Belgium), 1.1 µL of H_2_O (Nuclease free water), 0.5 μL of pooled *P. falciparum* and 0.4 μL of *P. malariae* primers and probes. The other species of *Plasmodium* were analyzed individually. Negative (PBS) and positive (DNA of each *Plasmodium* species) controls were used in all qPCR reactions. All assays were performed under standard conditions (1 cycle of 95 °C for 5 mins; 45 repeated cycles of 95 °C for 30 s and 60 °C for 30 s) with the CFX 96 Real-Time PCR machine (Bio-Rad Laboratories, Inc., Hercules, CA, USA). Samples with Ct higher than 38, which could suggest non-specific amplification, were excluded from subsequent analysis.

#### Entomological Parameters

The entomological parameters were calculated for the rainy and dry seasons following the formulas below in Table 2.

### 2.5. Identification of Anopheles Blood Meals

The ELISA procedure used in this study was adapted from Lardeux et al., with the modifications of using TMB (tetramethylbenzidine, Thermo Scientific, Rockford, IL, USA) substrate instead of orthotolidine for faster and more sensitive detection, the use of separate plates for each animal type to reduce cross-reactivity, and absorbance reading with a SpectraMax ABS plate reader (Molecular Devices, LLC, California, USA). All other steps were performed as previously described [13].

### 2.6. Data Analysis

Descriptive analyses were performed with R version 4.4.3. The “CrossTable” command of the “descry” library was used to compare *Anopheles* species according to season, as well as the gonotrophic states of *Anopheles* species per season. Fisher’s exact test and Pearson’s Chi-squared test were used depending on where they were applicable. Differences were considered statistically significant with *p* < 0.05. Prism version 10.1.2 was used for graphing.

## 3. Results

A total of 1082 and 1705 mosquitoes were caught during the rainy and dry seasons, respectively, inside the same houses. During the rainy season, 454 (41.9%) were *Anopheles*, 624 (57.7%) *Culex* and 4 (0.4%) *Aedes*. During the dry season, 65 (3.81%) were *Anopheles* and 1640 (96.19%) *Culex*. Morphologically, the anopheline population from Faladie consisted of 100% *An. gambiae s.l.* in the rainy and dry seasons. The principal malaria vectors found were in the *An. gambiae* complex across seasons. The abundance of malaria vectors peaked during the rainy season compared to the dry season. Out of the anopheline population, 412 (90.75%) were females and 42 (9.25%) were males during the rainy season, while during the dry season, 60 (92.30%) were females and 5 (7.70%) were males. The density of female *Anopheles* per room was 3.10 during the rainy season versus 0.30 during the dry season.

### 3.1. Anopheles Mosquito Species Diversity and Seasonal Abundance

**Figure 1:**
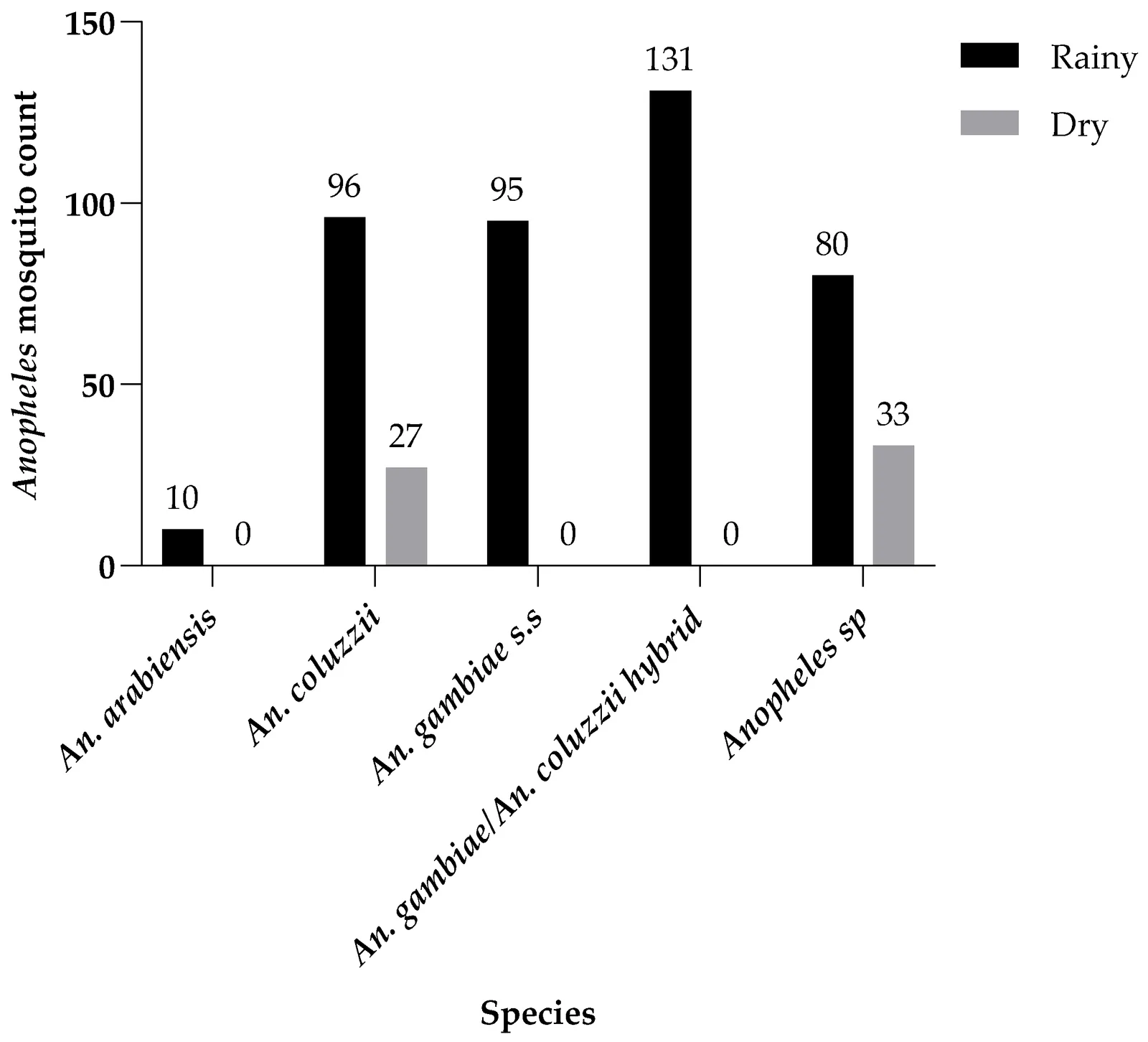
Composition and abundance of the *Anopheles* mosquito species in each season. The Y axis indicates the numbers of different *Anopheles* mosquito species. The X axis indicates different *Anopheles* mosquito species during the dry and rainy seasons. *An*: *Anopheles*. Black: *Anopheles* mosquitoes collected during the rainy season; grey: *Anopheles* mosquitoes collected during the dry season.

Of the 412 female *Anopheles* mosquitoes collected during the rainy season, 131 were identified as *An. gambiae/An. coluzzii* hybrids (31.8%), followed by *An. coluzzii* 96 (23.3%), *An. gambiae s.s.* 95 (23.1%) and *An. arabiensis* 10 (2.4%) (Figure 1, Table S1).

Representative gel images of the PRC-RFLP analysis for the different *Anopheles* species found in this study are shown in Figure S1.

Of the 60 female *Anopheles* mosquitoes collected during the dry season, 27 were identified as *An. coluzzii*, representing 45% of the sample (Figure 1, Table S1). The remaining *Anopheles* samples for all collections were not identified using the PCR-RLFP approach we applied. This failure of identification is attributed to poor DNA preservation.

*Anopheles arabiensis*, *An. gambiae s.s.* and the *An. gambiae/An. coluzzii* hybrid were found only during the rainy season and disappeared during the dry season. *An. coluzzii* was the only species found during the dry season. *Anopheles coluzzii* predominated during the dry season, with 45%. However, this species accounted for 23.3% of the *Anopheles* species collected during the rainy season. The *Anopheles gambiae/Anopheles coluzzii* hybrid was dominant during the rainy season, with 31.8%. *Anopheles gambiae* complex species composition and abundance differed significantly depending on the season according to Fisher’s exact test, *p* < 0.0001.

**Figure 2:**
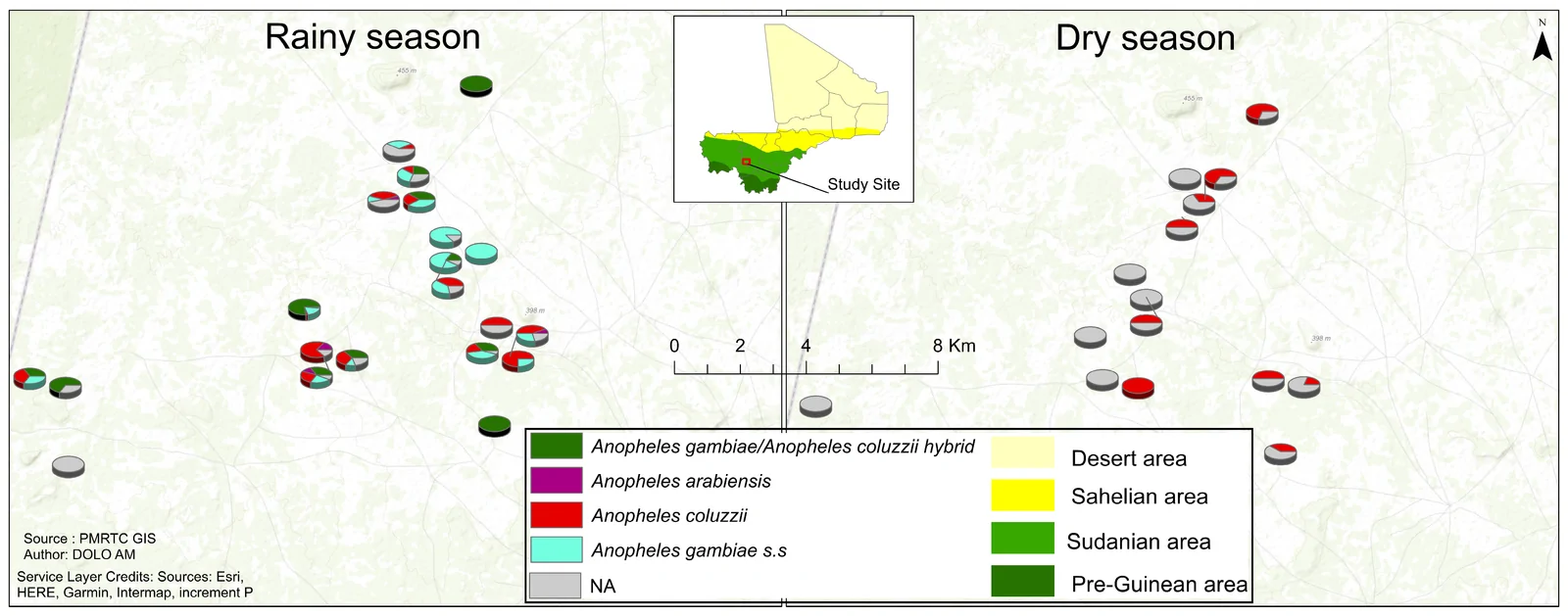
Seasonal distribution of the *Anopheles* mosquito species by concession in the Faladie area. This map shows the study site and the collection points. On the left is the distribution and frequencies of *Anopheles* mosquitoes during the rainy season, and on the right is the distribution and frequencies of *Anopheles* mosquitoes during the dry season. Samples were collected from the same houses. NA indicates that PCR failed to identify these *Anopheles* mosquitoes.

These results are supported by the mapping of the distribution of the *An. gambiae* complex species at the study site. Figure 2 shows the distribution of the *Anopheles* mosquito species across the collection points at the Faladie site. The small sectors represent the distribution and frequencies of each *Anopheles* species per collection point in the study area. These data include all mosquitoes collected during both the rainy and dry seasons in the same houses.

### 3.2. Plasmodium spp. Mosquito Infection Status and Entomological Inoculation Rates

All *An. gambiae s.l.* collected during both seasons were screened using a pan-*Plasmodium* qPCR assay. *Plasmodium falciparum* was the only species detected in mosquito heads/thorax samples. Out of the *Anopheles* mosquito species collected during the rainy season, 40 samples were positive. However, out of the *Anopheles* mosquito species collected during the dry season, only one sample was positive. *Anopheles gambiae* complex species infection status per season is described in Table 3. During the rainy season, 19 *An. gambiae/An. coluzzii* hybrids, 10 *An. coluzzii* and 6 *An. gambiae s.s.* were positive for *P. falciparum*, but no *An. arabiensis* specimens tested positive. The most infected species during the rainy season was the *An. gambiae/An. coluzzii* hybrid. But during the dry season, only one *An. coluzzii* species was infected by *P. falciparum*. For these collections, the overall infection rates for *P. falciparum* were 9.7% and 1.7% during the rainy and dry seasons, respectively (Table 3). Entomological inoculation rates during the rainy and dry seasons were 1.3095 and 0.0171, respectively. A significant difference in infectivity was observed between the rainy and dry seasons with Pearson’s Chi-squared test, with *p* = 0.032.

### 3.3. Female Gonotrophic State Composition by Season in Faladie

During the rainy season, blood-fed females *Anopheles* mosquitoes were the most abundant gonotrophic state, representing 48.5% of all females, followed by half-gravid (26.2%), unfed (21.7%) and gravid (3.6%) (Table 4, Figure S2). However, during the dry season, gravid female *Anopheles* mosquitoes dominated, comprising 40%, followed by half-gravid (33.3%), blood-fed (25%) and unfed (1.7%) (Table 4, Figure S2). No significant difference was observed in the female *Anopheles* gonotrophic state composition between seasons using Fisher’s exact test, *p* = 0.653. Table 4 summarizes the female *Anopheles* mosquito gonotrophic state composition for each season. The highest proportion of blood-fed females was observed in the *An. gambiae/An. coluzzii* hybrid during the rainy season.

### 3.4. Determination of Blood Meal Sources

**Figure 3:**
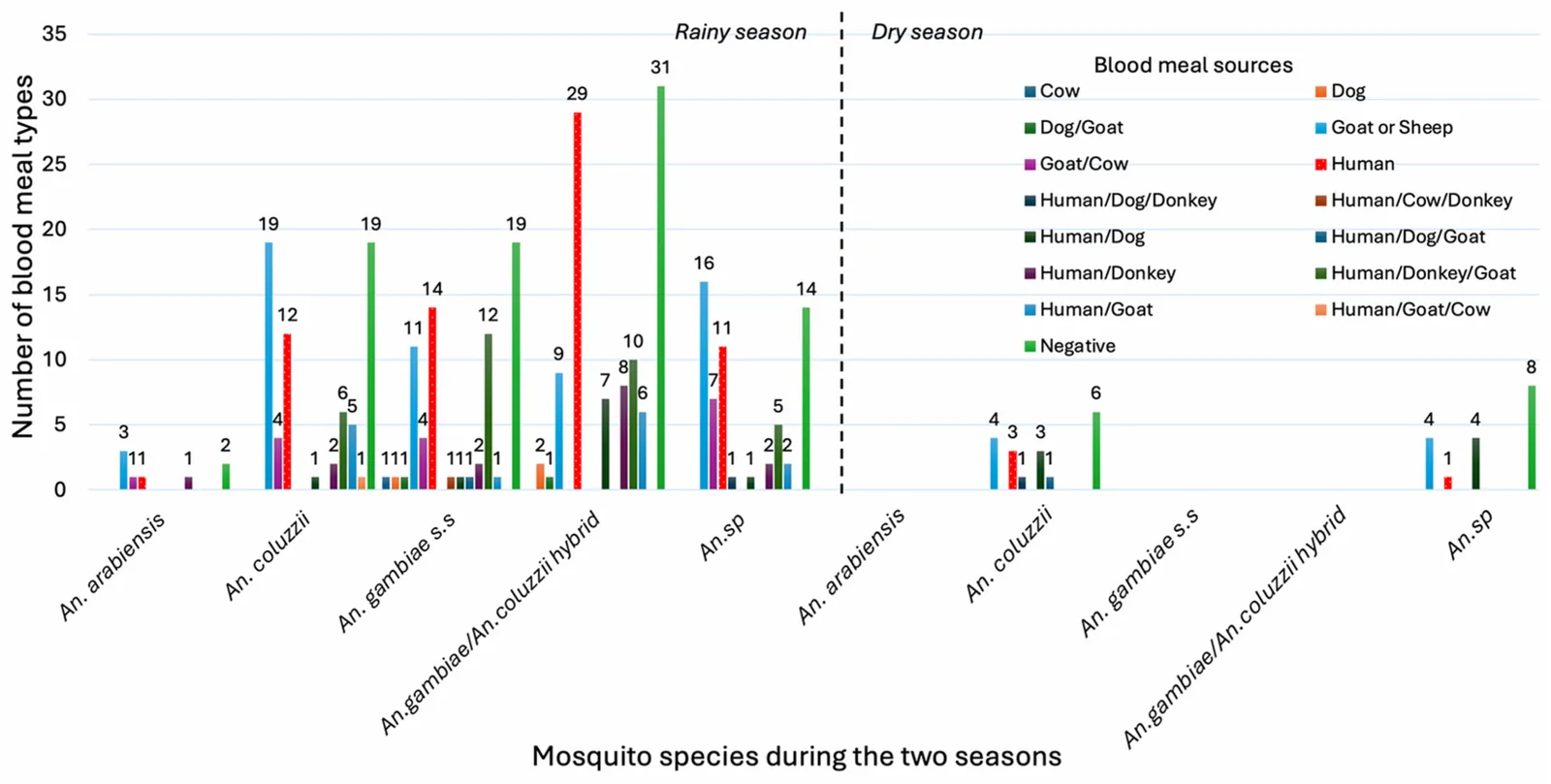
Blood meal sources. Human single meals are represented in red, with 8 mixed blood meals from animals and humans.

In this study, human blood meals dominated across seasons in the *An. gambiae s.l* mosquitoes. Animal blood meals and mixed meals were also observed among mosquitoes. The animal blood meal sources were goat or sheep, cow, donkey, dog and chicken (Figure 3, see Table S2). The total number of blood meals per category was calculated as the number of single meals + number of mixed meals/2. The *Anopheles gambiae s.l.* human blood meal index (HBI) corresponded to 71.5% during the rainy season. The *Anopheles gambiae/Anopheles coluzzii* hybrid had a higher rate of human blood meals, at 43.2%, than the other *Anopheles* mosquito species (Figure 3, see Table S2). *Anopheles coluzzii* carried significantly more animal blood meals compared to other *Anopheles* species. In comparison to *An. coluzzii* across seasons, animal-derived blood meals were significantly more likely during the rainy season than during the dry season. The proportion of human blood meals among *An*. *coluzzii* females was 18% (Figure 3, see Table S2). No significant difference was observed in the female *Anopheles* blood meal sources across seasons with Fisher’s exact test, *p* = 0.801.

## 4. Discussion

The dynamics, abundance and EIRs of malaria vector species in this study area are different during the two seasons. The principal malaria vectors identified in this study were within the *An. gambiae* complex *(An. gambiae s.s.*, *An. coluzzii* and *An. arabiensis),* followed by the *An. gambiae/An. coluzzii* hybrid. This is consistent with other studies, particularly in the Sahelian region of Mali [8,14,15]. The composition of *An. gambiae* complex species during the rainy season comprised the *An. coluzzii*, *An. gambiae s.s.*, *An. gambiae/An. coluzzii* hybrid and *An. arabiensis.* A high abundance of these mosquitoes was observed during the rainy season. However, only *An. coluzzii* remained dominant during the dry season, as also reported in previous study by Dao et al. [8]. This species could sustain local transmission of malaria during the dry season.

In the Faladie area, *P. falciparum* was the only malaria parasite species found in the mosquitoes captured during the rainy and dry seasons, accounting for 9.7% and 1.7%, respectively.

Significant differences in EIRs were observed during the rainy and dry seasons, with 1.3095 and 0.0171, respectively. A significant difference was observed in infectivity between the rainy and dry seasons using Pearson’s Chi-squared test (*p* = 0.032).

During the rainy season, blood-fed female *Anopheles* mosquitoes were the most abundant gonotrophic state, representing 48.5% of all females, followed by half-gravid (26.2%). This high proportion of blood-fed *An. gambiae s.l.* during the rainy season indicates active blood feeding during this period versus during the dry season. Meanwhile, during the dry season, gravid female *Anopheles* mosquitoes dominated, at 40%, followed by half-gravid (33.3%). These results are different from those of Yaro et al., who demonstrated seasonal variation in *An. coluzzii,* with the lowest blood-feeding response occurring during the rainy season [15]. The behaviors of each *Anopheles* mosquito species differ, particularly regarding their host preference and blood-feeding behavior. Better understanding of these behaviors is crucial for effective malaria control strategies [16,17]. In this area during the rainy season, the human blood meal index (HBI) of *An. gambiae s.l.* corresponded to 71.5%. Particularly, the *An. gambiae/An. coluzzii* hybrid had a higher rate of human blood meals (43.2%) than other *Anopheles* mosquito species. The proportion of human blood meals by *An*. *coluzzii* females was 18%. In comparison to *An. coluzzii* across seasons, animal blood meal sources were significantly more likely during the rainy season than during the dry season. These findings suggest that *An. gambiae s.l.* are both anthropophilic and zoophilic at this study site. Animal blood meals are due to opportunistic behavior adopted by mosquitoes to complete their blood meals [7,16]. They only need to bite an infected human during their first blood meal and then survive long enough to bite another human once they become infectious to transmit the parasites [18,19]. This behavior enables them to evade control measures, resulting in a higher survival rate and increased vector competence for malaria transmission. Despite the numerous control measures used by the local population, malaria transmission continues in this village. These control measures are primarily implemented to prevent transmission from humans to the vector through artemisinin-based combination therapies (ACTs), intermittent preventive treatment of pregnant women (IPTp), seasonal malaria chemoprevention (SMC) [20], and the active and passive detection of cases through numerous studies [3]. Measures to prevent transmission from the vector to humans, notably indoor residual spraying (IRS), long-lasting insecticide-treated mosquito nets (LLINs) and larval source management, are not being effectively implemented in this area. Control of adult mosquitoes and the implementation of community participation and education will help to reduce the rate of malaria transmission in this area. In addition, the zoophilic tendency of *An. gambiae s.l.* in Faladie remains worrying and could increase the risk of them transmitting zoonotic diseases [18]. Indeed, with the emergence of arboviruses in Mali and the current outbreak of Rift Valley fever (RVF) in Senegal and Mauritania, two countries that border Mali [21,22,23], it is important to screen malaria vector *An. gambiae s.l.* for their potential transmission of these arboviruses.

## 5. Conclusions

This study provides better understanding of the seasonal dynamics of malaria vectors circulating in Faladie. The vectors found were mostly members of the *An. gambiae* complex, such as *An. coluzzii*, *An. gambiae s.s.*, *An. arabiensis* and a high proportion of the *An. gambiae/An. coluzzii* hybrid. These vectors and malaria transmission dynamics follow seasonal variations, and the highest mosquito abundance and EIRs occur during the rainy season and decrease significantly during the dry season. However, despite low residual mosquito population, those infected with malaria parasites remained present during the dry season. *Anopheles coluzzii* species can survive during both rainy and dry seasons and sustain malaria transmission. This study highlights the importance of strategically tackling residual dry season malaria transmission to control the disease in rural areas of Mali and thus move toward eradication.

## Figures and Tables

**Table 1:** Primers and probes used for identification of *Plasmodium* species.

Species	Primer or Probe	Sequence	Reference
*P. falciparum*	Pf-F	CCGACTAGGTGTTGGATGAAAGTGTTAA	[11]
*P. falciparum*	Pf-R	AACCCAAAGACTTTGATTTCTCATAA	[11]
*P. falciparum*	Pf-Probe	CY5-AGCAATCTAAAAGTCACCTCGAAAGATGAC	[11]
*P. malariae*	Pm-F	CCGACTAGGTGTTGGATGATAGAGTAAA	[11]
*P. malariae*	Pm-R	AACCCAAAGACTTTGATTTCTCATAA	[11]
*P. malariae*	Pm-Probe	FAM-CTATCTAAAAGAAACACTCAT	[11]
*P. vivax*	Pv-F	CCGACTAGGCTTTGGATGAAAGATTTT	[11]
*P. vivax*	Pv-R	AACCCAAAGACTTTGATTTCTCATAA	[11]
*P. vivax*	Pv-Probe	FAM-AGCAATCTAAGAATAAACTCCGAAGAGAAA	[11]
*P. ovale curtisi*	OVAC-F	TTTTGAAGAATACATTAGGATACAATTAATG	[12]
*P. ovale curtisi*	OVAC-R	CATCGTTCCTCTAAGAAGCTTTACAAT	[12]
*P. ovale curtisi*	OVAC-Probe	HEX-CCTTTTCCCTATTCTACTTAATTCGCAATTCATG	[12]
*P. ovale wallikeri*	OVAW-F	TTTTGAAGAATATATTAGGATACATTATAG	[12]
*P. ovale wallikeri*	OVAW-R	CATCGTTCCTCTAAGAAGCTTTACAAT	[12]
*P. ovale wallikeri*	OVAW-Probe	FAM-CCTTTTCCCTTTTCTACTTAATTCGCTATTCATG	[12]

**Table 2:** Entomological parameters for malaria transmission.

Parameters	Formulas
Densities of female mosquitoes per room	Total number of collected mosquitoes/total number of rooms
Sporozoite infection rate in mosquitoes	(Total positive in qPCR/total tested) × 100
Human biting rates (HBRs)	Total number of freshly fed mosquitoes/number of residents that have slept in the rooms the previous night
Entomological inoculation rates (EIRs)	HBR × (total positive in qPCR/total tested)

**Table 3:** *Anopheles gambiae* complex species *Plasmodium falciparum* infection status by season.

**Season**	**Species**	**Negative**	**Positive**
		*n* (%)	*n* (%)
Rainy	*An. gambiae s.s.*	89 (24)	6 (15)
	*An. coluzzii*	86 (23.1)	10 (25)
	*An. arabiensis*	10 (2.7)	0 (0.0)
	*An. gambiae/An. coluzzii* hybrid	112 (30.1)	19 (47.5)
	*An.* sp.	75 (20.1)	5 (12.5)
Total		372 (90.3)	40 (9.7)
Dry	*An. coluzzii*	26 (44)	1 (100)
	*An.* sp.	33 (56)	0 (0)
Total		59 (98.3)	1 (1.7)

*An*: *Anopheles*, Pearson’s Chi-squared test, *p* = 0.032.

**Table 4:** Gonotrophic states of *Anopheles gambiae* complex species by season.

**Season**	**Species**	**Blood-Fed**	**Half-Gravid**	**Gravid**	**Unfed**	**Total**
		*n* (%)	*n* (%)	*n* (%)	*n* (%)	*n* (%)
Rainy	*An. gambiae s.s.*	44 (22)	25 (23.1)	3 (20)	23 (25.9)	95 (23.1)
	*An. coluzzii*	46 (23)	23 (21.3)	3 (20)	24 (27)	96 (23.3)
	*An. arabiensis*	5 (2.5)	3 (2.8)	0 (0.0)	2 (2.2)	10 (2.4)
	*An. gambiae/An. coluzzii* hybrid	70 (35)	33 (30.6)	1 (6.7)	27 (30.3)	131 (31.8)
	*An.* sp.	35 (17.5)	24 (22.2)	8 (53.3)	13 (14.6)	80 (19.4)
Total		200 (48.5)	108 (26.2)	15 (3.6)	89 (21.7)	412 (100)
Dry	*An. coluzzii*	8 (53.3)	10 (50)	9 (37.5)	0 (0.0)	27 (45)
	*An.* sp.	7 (46.7)	10 (50)	15 (62.5)	1 (100)	33 (55)
Total		15 (25)	20 (33.3)	24 (40)	1 (1.7)	60 (100)

*An: Anopheles*, Fisher’s exact test, *p* = 0.653.

## Data Availability

All data generated are included in this published article and its Supplementary Materials.

## Supplementary Materials

The following supporting information can be downloaded at https://www.mdpi.com/article/10.3390/tropicalmed11080216/s1: Table S1. Composition and abundance of *Anopheles gambiae* complex species in Faladie by season; Figure S1. Representative gel images for PCR-RFLP of different *Anopheles* species found in this study. L: 1 kb Ladder size standard, AG: *Anopheles gambiae s.s.*, AC: *Anopheles coluzzii*, AR: *Anopheles arabiensis,* GC: *Anopheles gambiae*/*Anopheles coluzzii hybrid;* Figure S2. Gonotrophic states of *Anopheles* mosquito species across season. The Y axis indicates different gonotrophic states including blood-fed, half-gravid, gravid and unfed. The X axis indicates seasonality (rainy and dry seasons); Table S2. *Anopheles gambiae* complex species of blood meal sources in Faladie across seasons.
