# Supplementary material for: Malaria Vectors’ Diversity and Seasonality in Sustaining Disease Transmission in an Endemic Area of Faladie, Mali
Source: Trop Med Infect Dis. 2026 Aug 3;11(8):216. doi: 10.3390/tropicalmed11080216 (PMC13517914; doi:10.3390/tropicalmed11080216)
Supplement: Supplementary file 1 [file tropicalmed-11-00216-s001.zip › tropicalmed-4359794-supplementary.pdf]

Supplementary Materials:

Table S1. Composition and abundance of *Anopheles gambiae* complex species in Faladie by season.

Species/season	Rainy *n* (%)	Dry *n* (%)	Total
An. gambiae s.s.	95 (23.1)	0 (0)	95
An. coluzzii	96 (23.3)	27(45)	123
An. arabiensis	10 (2.4)	0 (0)	10
An. gambiae/An. coluzzii hybrid	131 (31.8)	0 (0)	131
An. sp.	80 (19.4)	33 (55)	113
Total	412 (100)	60 (100)	472

An: *Anopheles,* Fisher's Exact Test, $p < 0.0001$

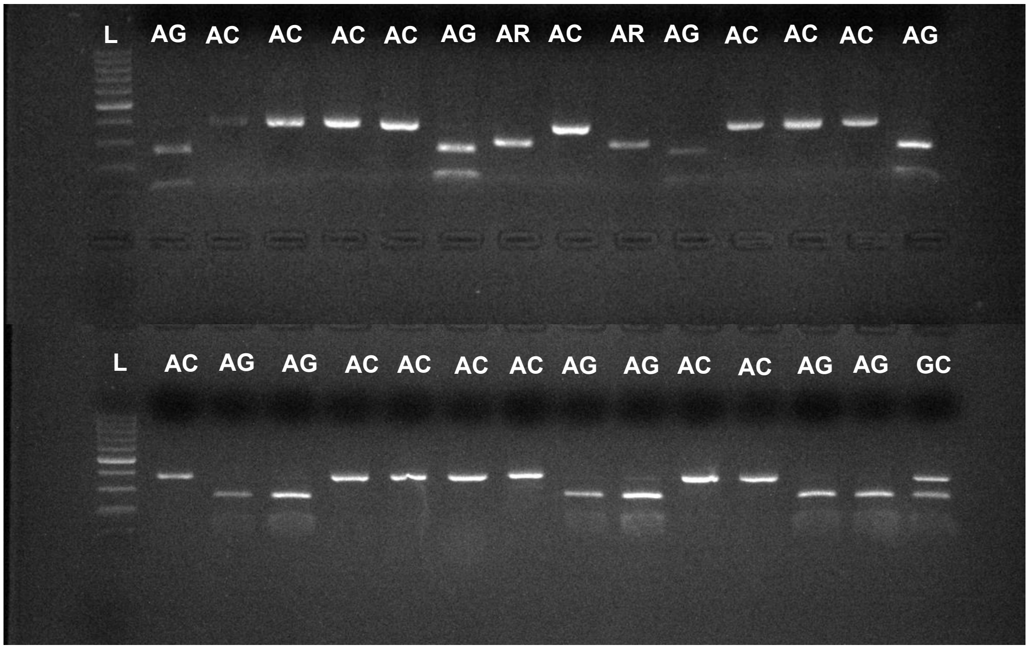

Figure S1. Representative gel images for PCR-RFLP of different *Anopheles* species found in this study, L: 1 kb Ladder size standard, AG: *Anopheles gambiae s.s.,* AC: *Anopheles coluzzii,* AR: *Anopheles arabiensis,* GC: *Anopheles gambiae/ Anopheles coluzzii hybrid.*

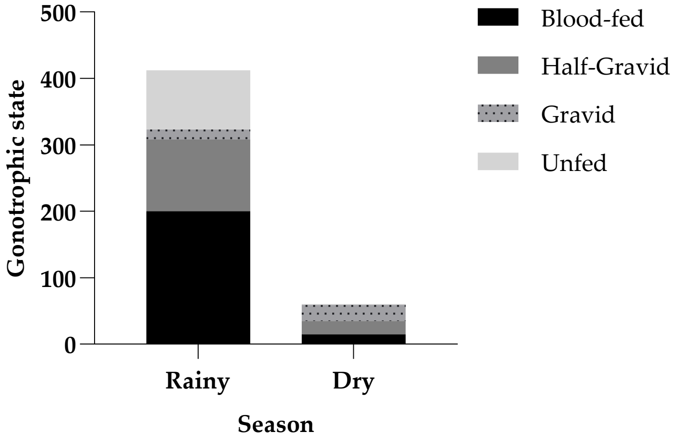

Figure S2. Gonotrophic states of *Anopheles* mosquito species across season. Y Axis: indicate different gonotrophic states including Blood-fed, Half-gravid, Gravid and Unfed and X Axis: indicate seasonality (rainy and dry seasons).

Table S2. *Anopheles gambiae* complex species of blood meal sources in Faladie across season (N=308 rainy and 35 dry).

**Season**	**Species**	Human	Cow	Dog	Goat or Sheep	Human / Goat	Human /Donkey	Human /Dog	Dog/Goat	Cow/ Goat	Human /Cow/Donkey	Human /Goat/Cow	Human/ Donkey/ Goat	Human / Dog/ Donkey	Human /Dog/ Goat	Negative
		n (%)	*n* (%)	*n* (%)	*n* (%)	*n* (%)	*n* (%)	*n* (%)	*n* (%)	*n* (%)	*n* (%)	*n* (%)	*n* (%)	*n* (%)	*n* (%)	*n* (%)
Rainy	*An. gambiae s.s.*	14 (20.9)	1 (100)	1 (33.3)	11 (18.95)	1 (7.15)	2 (13.3)	1 (10)	1 (50)	4 (25)	1(100)	0 (0.0)	12 (36.36)	0 (0.0)	1 (100)	19 (22.35)
	An. coluzzii	12 (18)	0 (0.0)	0 (0.0)	19 (32.76)	5 (35.7)	2 (13.3)	1 (10)	0 (0.0)	4 (25)	0 (0.0)	1(100)	6 (18.19)	0 (0.0)	0 (0.0)	19 (22.35)
	An. arabiensis	1 (1.5)	0 (0.0)	0 (0.0)	3 (5.17)	0 (0.0)	1 (6.7)	0 (0.0)	0 (0.0)	1 (6.25)	0 (0.0)	0 (0.0)	0 (0.0)	0 (0.0)	0 (0.0)	2 (2.3)
	An. gambiae/An. coluzzii hybrid	29 (43.2)	0 (0.0)	2 (66.7)	9 (15.52)	6 (42.85)	8 (53.4)	7 (70)	1 (50)	0 (0.0)	0 (0.0)	0 (0.0)	10 (30.3)	0 (0.0)	0 (0.0)	31 (36.5)
	An. sp.	11 (16.4)	0 (0.0)	0 (0.0)	16 (27.6)	2 (14.3)	2 (13.3)	1 (10)	0 (0.0)	7 (43.75)	0 (0.0)	0 (0.0)	5 (15.15)	1 (100)	0 (0.0)	14 (16.5)
Total		67	1	3	58	14	15	10	2	16	1	1	33	1	1	85
Dry	*An. coluzzii*	3 (75)	0 (0.0)	0 (0.0)	4 (50)	0 (0.0)	0 (0.0)	3 (43)	0 (0.0)	0 (0.0)	0 (0.0)	0 (0.0)	0 (0.0)	1 (100)	1 (100)	6 (43)
	An. sp.	1 (25)	0 (0.0)	0 (0.0)	4 (50)	0 (0.0)	0 (0.0)	4 (57)	0 (0.0)	0(0.0)	0 (0.0)	0 (0.0)	0 (0.0)	0 (0.0)	0 (0.0)	8 (57)
Total		4	0	0	8	0	0	7	0	0	0	0	0	1	1	14

An: Anopheles, Fisher's Exact Test, $p = 0.801$.
